# Production of potent antimicrobial agent by actinomycete, *Streptomyces sannanensis* strain SU118 isolated from phoomdi in Loktak Lake of Manipur, India

**DOI:** 10.1186/s12866-014-0278-3

**Published:** 2014-11-19

**Authors:** Laishram Shantikumar Singh, Hemant Sharma, Narayan Chandra Talukdar

**Affiliations:** Institute of Bioresources and Sustainable Development, Sikkim Centre, DBT, Tadong, Gangtok, 737102, Sikkim India; Institute of Bioresources and Sustainable Development, DBT, Takyelpat Institutional Area, Imphal, 795001, Manipur India

**Keywords:** Phoomdi, *Streptomyces sannanensis* SU118, Antibacterial potential, Minimum inhibitory concentration

## Abstract

**Background:**

Actinomycetes have provided a wealth of bioactive secondary metabolites with interesting activities such as antimicrobial, antiviral and anticancer. The study aims at isolation, characterization and the antimicrobial potentiality of *Streptomyces sannanensis* SU118 obtained from *Phoomdi,* a unique habitat of Loktak Lake of Manipur, India.

**Results:**

An actinomycete strain isolated from *Phoomdi* soil of Loktak Lake of Manipur, India was identified as *Streptomyces sannanensis* SU118. It is a Gram-positive filamentous bacterium which exhibits antimicrobial activity only against Gram-positive bacteria, while Gram-negative organisms were not affected. Glucose Soyabean meal broth was found to be the suitable medium for antibiotic production at 28°C for seven days of incubation. The antimicrobial agent produced by the strain was extracted with ethyl acetate as solvent and purified by thin layer chromatography. Screening and bioassay - guided fractionation of the ethyl acetate extract from the culture filtrate led to the isolation of an active potential compound (*R*_*f*_ value 0.56) with λ_max_ 275.0 nm which has got the lowest minimum inhibitory concentration (0.5 μg/ml) against *Staphylococcus aureus* MTCC 96 and *Staphylococcus aureus* (clinical isolate), whereas highest (3.0 μg/ml) was recorded against *Mycobacterium smegmatis* MTCC 6 and *Bacillus circulans* MTCC 8074.

**Conclusion:**

This study has therefore uncovered the potential of exploring virgin untapped habitats in the Indo-Burma biodiversity hot spot region as reservoir of promising antimicrobial metabolite producer. These results highlighted the scope for further characterization of the metabolite and could be a candidate in the generation of new antimicrobial agents.

## Background

Microbial natural products are the origin of most of the antibiotics on the market today. There is an alarming scarcity of new antibiotics currently under development in the pharmaceutical industry. Still, microbial natural products remain the most promising source of novel antibiotics, although new approaches are required to improve the efficiency of the discovery process. Actinomycetes have provided important bioactive compounds of high commercial value and continue to be routinely screened for new bioactive substances [1,2]. It is generally accepted that the streptomycetes have a particular capacity to produce a large variety of different bioactive compounds that have a wide spectrum of activity [3]. It is reported that 45% of the presently known bioactive microbial metabolites were still isolated from various actinomycetales species, and the *Streptomyces* species produces 7600 compounds (74% of all actinomycetales), while the rare actinomycetes represent 26%, altogether 2500 compounds [4]. These organisms produce perhaps the most diverse and most unique, unprecedented, sometimes very complicated compounds exhibiting excellent antimicrobial potency and usually low toxicity [4]. The metabolic diversity of the actinomycetes is due to its extremely large genome, which has hundreds of transcription factors that control gene expression, allowing them to respond to specific needs [5]. *Streptomyces* sp. strain 201 endowed with novel antibiotic property has been isolated from tea garden soil [6]. Antimicrobial potentiality of salt-tolerant and alkaliphilic strain, *Streptomyces tanashiensis* A2D has also been reported by Singh *et al*. [7]. A new antiherpetic agent, fattiviracin has been isolated from *Streptomyces microflavus* [8]. These recent examples from the literature highlights the fact that despite extensive exploration of the actinomycetes for their antimicrobial products in the past, the search for novel molecules having unique therapeutic properties continues to be an active area of research. In the course of screening for new antibiotics, several studies are oriented towards isolation of new *Streptomyces* species from different habitats. Although soils have been screened by many workers for the past many decades, only a small fraction of actinomycetes have been discovered [9]. Actinomycetes from unexplored habitats have gained considerable attention in recent years for the production of bioactive metabolites.

The expanding list of novel microorganisms and the products derived from poorly explored areas of the world like Jordan [10], Antarctica [11] and certain biotope of Manipur [7] suggest that a careful exploration of new habitats might continue to be useful. The isolation of these microorganisms that produce bioactive compounds is of great interest in the development of new molecules to fight against many pathogen especially with the emergence of antibiotic multi-resistant pathogens.

The present study is a part of our on-going and extensive screening being carried out in the hitherto unexplored habitats of North-East India, under Indo-Burma Biodiversity hot spot with a view to realize the bioresources and value they have to mankind. This communication deals with the isolation, characterization and antimicrobial potentiality of a new actinomycete strain identified as *Streptomyces sannanensis* SU118.

## Methods

### Location and collection of soil sample

Soil samples were collected at a depth of 10–15 cm from the upper surface of top soil from *phoomdi* in Loktak lake (latitude, 24^0^ 25′ - 24^0^ 42′ N; longitude, 93^0^ 46′ - 93^0^ 55′ E) of Manipur, which is the largest natural lake (289 Km^2^) in eastern India, where a unique ecological condition prevails. *Phoomdi* (Manipuri word meaning floating mats of soil and vegetation) is a unique untapped habitat with heterogenous mass of soil, vegetation and organic matter in different stages of decay. Seven soil samples each of approximately 50 g were collected from the pristine site. Each sampling was carried out at least 5 m apart. The samples were placed in sterile plastic containers, which were tightly sealed and transported to the laboratory.

### Culture media

Starch casein agar (SCA): Soluble starch, 10.0 g; casein, 0.3 g; KNO_3_, 2.0 g; NaCl, 2.0 g; K_2_HPO_4_, 2.0 g; MgSO_4_.7H_2_O, 0.05 g; CaCO_3_, 0.02 g; FeSO_4_.7H_2_O, 0.01 g; Agar, 20.0 g; distilled water, 1,000 ml; pH 7.0. Actinomycetes Isolation Agar {(AIA); HiMedia Laboratories Pvt. Limited, Mumbai, India}: Sodium caseinate, 2.0 g; Asparagine, 0.1 g; Sodium propionate, 4.0 g; Dipotassium phosphate, 0.5 g; Magnesium sulphate, 0.1 g; Ferrous sulphate, 0.001 g and Agar 20.0 g; distilled water, 1,000 ml; pH 7.0. Glucose Soyabean meal broth (GSB) containing Glucose, 10.0 g; Soyabean meal, 10.0 g; NaCl, 10.0 g; CaCO_3,_ 1.0 g; distilled water, 1,000 ml and pH adjusted to 7.0 was used as the production medium.

### Test microorganisms

The test bacterial strains used for screening of antimicrobial activity were procured from microbial type culture collection (MTCC) and Gene Bank of the Institute of Microbial Technology (IMTECH), Chandigarh, India and are: *Bacillus subtilis* MTCC 736, *Bacillus circulans* MTCC 8074, *Bacillus megaterium* MTCC 8075, *Staphylococcus aureus* MTCC 96*, Micrococcus luteus* MTCC 2987, *Mycobacterium phlei* MTCC 1724, *Mycobacterium smegmatis* MTCC 6, *Escherichia coli* MTCC 739, *Klebsiela pneumoniae* MTCC 3040, *Pseudomonas aeruginosa* MTCC 2453. Apart from these one clinical isolate of *Staphylococcus aureus* obtained from Down Town Hospital Ltd., Guwahati, Assam, India was also used as test pathogen during the investigation.

### Isolation of the actinomycete strain

During the course of screening for antibiotics from actinomycetes of Loktak habitats, the strain SU118 was isolated by serial dilution technique using SCA medium [12] by incubating at 28°C for 10 days from the soil sample collected from *phoomdi* in Loktak lake of Manipur, India. The pure culture of the isolate was obtained by repeated streaking on SCA plates. The pure isolate was transferred to SCA slants and preserved at 4°C for further use.

### Identification of the actinomycete strain

The identification of the strain SU118 was carried out on the basis of the cultural characteristics, morphological, biochemical characteristics, through the courtesy of MTCC and Gene Bank of IMTECH, Chandigarh, India and also by the 16S rRNA gene sequencing carried out through the courtesy of National Centre for Cell Science, Pune, India.

### Construction of phylogenetic tree

The sequenced 16S rRNA gene of the strain SU118 was aligned with the nucleotide sequences of *Streptomyces* genera in GenBank database using BLAST [13]. Sequences with more than 98% homology were considered for molecular taxonomy analysis. Multiple alignments of 16S rRNA nucleotide sequences in this study and sequences from GenBank database was performed with CLUSTAL W program [14]. A phylogenetic tree was generated using neighbor-joining method [15] with bootstrap testing [16] of 1,000 replicates, in MEGA6 [17].

### Morphological, cultural, physiological and biochemical characteristics

The morphological characteristics were studied by using cover slip method in which the culture was transferred to the base of cover slips buried in SCA medium [18] and incubated at 28°C for seven days. The morphology of spore bearing hyphae with entire spore chain was then studied as described in Bergey’s manual [19]. Carbohydrate utilization was determined by growth on carbon utilisation medium (International Streptomyces Project 9) [20] supplemented with 1% carbon source at 28°C. Temperature range, pH range and NaCl tolerance for growth was determined on inorganic salt starch agar medium (ISP 4) by growing at different temperatures (20–42°C), pH (5–10) and NaCl concentration (1-5% NaCl) respectively. Hydrolysis of starch, Liquefaction of gelatin and other biochemical tests were evaluated by following the methods of Gordon *et al*. [21]. Reduction of nitrate and production of melanoid pigment were determined by the method of ISP [22]. Determination of LL-Diaminopimelic acid (L-DAP) and sugar pattern were carried out according to Becker *et al*. [23] and Lechevalier and Lechevalier [24]. All the results were recorded after seven days of incubation.

### Determination of antimicrobial potential of strain SU118

#### Spot inoculation on agar medium

The antimicrobial activity was studied primarily by spot inoculation technique on agar medium as reported by Singh *et al*. [12]. The isolate SU118 was spot inoculated on SCA, the petri plates were incubated at 28°C for seven days and then inverted for 40 minutes over chloroform in fumehood. Colonies were then covered with a 0.6% agar layer of nutrient agar (NA) previously seeded with bacterial suspension of 1.5 × 10^8^ colony forming units (CFU/spores)/ml in sterile normal saline [25] of the target bacterial strains and then incubated at 37°C for 24 h. The zone of inhibition of growth of the test microorganisms around the actinomycete isolate SU118 was observed after incubation. *S. aureus* MTCC 96 was more sensitive to the antimicrobial agent; hence further studies were carried out with it.

### Antimicrobial agent production in liquid medium

After preliminary testing for its antimicrobial potentiality, the isolate SU118 was further studied for the production of antimicrobial agent in liquid medium in shake flask condition. The effect of culture media on production of antimicrobial agent was examined by taking different liquid media such as Starch casein broth, Actinomycetes broth, Nutrient broth and GSB. A homogenous bacterial suspension (0.2 O.D measure at 600 nm) of the isolate SU118 in 0.05% Tween 20 was prepared by taking inoculum from seven days old culture grown on SCA plate at 28°C. Five ml of this suspension was inoculated into 100 ml of each liquid media contained in 250 ml Erlenmeyer flask and incubated at 28°C in a shaking incubator maintained at 150 rpm for seven days. After incubation each culture broths were centrifuged at 10,000 × g (Sorvall Biofuge Primo R) for 20 minutes at room temperature. The cell free supernatants were tested for extracellular antimicrobial activity by standard agar well diffusion method [26] against *S. aureus* MTCC 96, the most sensitive test organism in preliminary screening. Nutrient agar plates were inoculated with 0.2 ml of overnight culture of *S. aureus* suspension containing 1.5 × 10^8^ cells as mentioned above and uniformly spread out with the help of sterile glass spreader. Agar wells (6 × 4 mm) were prepared by scooping out the medium with a sterile cork borer. The supernatant culture broth of each medium were then administered to the wells separately and incubated at 37°C for 24 h. Antimicrobial activity was determined by measuring the inhibition zone diameter (in mm) of *S. aureus* MTCC 96 around the well after incubation. Each experiment was conducted in three replicates and the mean value of inhibition zone diameter was calculated. Uninoculated broth medium added to the wells were taken as control. Among the different liquid media tried, GSB was found to be the best medium for antimicrobial agent production by the isolate SU118; hence GSB was used as production medium for further studies.

### Effect of temperature, pH and incubation period on growth and production of antimicrobial agent

The effect of temperature on growth and production of antimicrobial agent was studied on GSB at different temperatures (25, 28, 31, 34 and 37°C) at pH 7. Five ml of spore suspension of isolate SU118 was inoculated into 100 ml of GSB contained in 250 ml Erlenmeyer flask and incubated in a shaking incubator maintained at 150 rpm for seven days. The antimicrobial activity was evaluated against *S. aureus* MTCC 96 by agar well diffusion method in the similar manner as mentioned above. Similarly, the effect of pH on the production of antimicrobial agent was studied at different pH (5–9) using GSB by incubating at 28°C for seven days. The effect of incubation period on the production of antimicrobial agent was also studied in the similar way as above by incubating for different days (1–10) at 28°C using GSB at pH 7.

### Estimation of growth

The biomass from the culture filtrate separated by means of centrifugation was transferred to a pre-weighed dry filter paper using a clean spatula and then placed in an oven at 50°C overnight to reach a fixed weight. Growth in terms of biomass accumulation was expressed as mg/ml of culture medium.

### Specific rate of product formation (qp)

The specific rate of production of the antimicrobial agent (qp) was calculated according to the following equation:

$$ \mathrm{q}\mathrm{p}=1/\mathrm{X}\left(\mathrm{d}\mathrm{p}/\mathrm{d}\mathrm{t}\right), $$

Where X is the biomass concentration (mg/ml), p is antimicrobial agent concentration and t is time respectively. The derivative dp/dt was calculated according to the method proposed by Le Duy and Zajic [27].

### Extraction, purification and bioassay of the metabolite

The culture broth of the isolate SU118 grown on GSB for 7 days at 28°C in a shaking incubator (150 rpm) was centrifuged at 10,000 × g (Sorvall Biofuge Primo R) for 20 minutes at room temperature. The crude bioactive metabolite produced in liquid culture medium by the isolate SU118 was extracted from the supernatant obtained by manual shaking thrice with equal volume of ethyl acetate (1:1) in a separating funnel. The solvent layer was collected and then evaporated under vacuum in a rotary evaporator (Buchi R-114, Germany) maintained at 40°C water bath and the crude metabolite thus obtained was subjected to partial purification. Purification of this crude metabolite was carried out by thin layer chromatography (TLC) technique on silica gel (Merck Ltd.) using hexane-ethyl acetate (Merck Ltd.) gradient (1:1 → 1:3) as running solvent system. TLC purified fractions were recovered and antimicrobial activity was tested by agar well diffusion method against all the test microorganisms to find out the active metabolite among the different fractions. Each fraction was dissolved in 10% dimethyl sulfoxide (DMSO) to get a concentration of 1 mg/ml. From this 100 μl were loaded in the agar wells prepared on the microbial bioassay plates as mention above. Wells filled with 10% DMSO solution served as control. The Petri plates were incubated at 37°C for 24 h. The inhibition zone diameter against the test microorganisms was recorded. Three replicates were maintained in each case.

### Minimum inhibitory concentration (MIC)

MIC was determined according to Boruwa *et al*. [28] by adding an inoculum size of 3 × 10^5^ colony forming units (cfu)/ml of test microorganisms to 5 ml of nutrient broth in different test tubes. Serial dilutions of the active metabolite (concentration ranging from 10 μg to 0.25 μg/ml) were added at the same time separately in the respective tube. MIC of the active metabolite against the bacterial test organisms was determined after 48 h of incubation by removing 10 μl of the contents from each tube and spreading them onto NA plates. Each set of experiment was carried out in three replicates. Growth of the test microorganisms were observed after 24 h of incubation at 37°C. In the similar way MIC of known antibiotic penicillin was also carried out as a standard for comparison. MIC is defined as the lowest concentration required to inhibit any visible growth.

### Determination of rate of kill

*In vitro* assays for the rate of killing bacteria by the antimicrobial agent were carried out using a modified plating technique of Eliopoulos and Eliopoulos [29] and Eliopoulos and Moellering [30]. The antimicrobial agent was incorporated into 10 ml Mueller Hinton broth in McCartney bottles at 1/2 MIC, 1 × MIC, and 2 × MIC. Two controls, one Mueller Hinton broth without the antimicrobial agent inoculated with test organisms and Mueller Hinton broth incorporated with the antimicrobial agent at the test concentrations without the test organisms, were included. Inoculums density, approximately 3 × 10^5^ cfu/ml further verified by total viable count, was used to inoculate 10 ml volumes of both test and control bottles. The bottles were incubated at 37°C on an orbital shaker at 120 rpm. A 100 μl aliquot was removed from the culture medium at 0, 4, and 8 h for the determination of cfu/ml by the plate count technique [31] by plating out 25 μl of each of the dilutions. The problem of antimicrobial agent carryover was addressed by dilution as described previously by Pankuch *et al*. [32]. After incubating at 37°C for 24 h, emergent bacterial colonies were counted, cfu/ml calculated, and compared with the count of the culture control without the antimicrobial agent. Each experiment was performed in duplicate and mean value obtained. The results were expressed as negative or positive log_10_ values according to Baltch *et al*. [33].

### Statistical analysis

Statistical analysis was carried out by calculating the means and standard deviations of the results. Duncans multiple range test (DMRT) was done to compare that the sample means were significantly different from each other at a significant level of P >0.001 [34].

## Results

### Characterization and taxonomy of the strain

**Figure 1:**
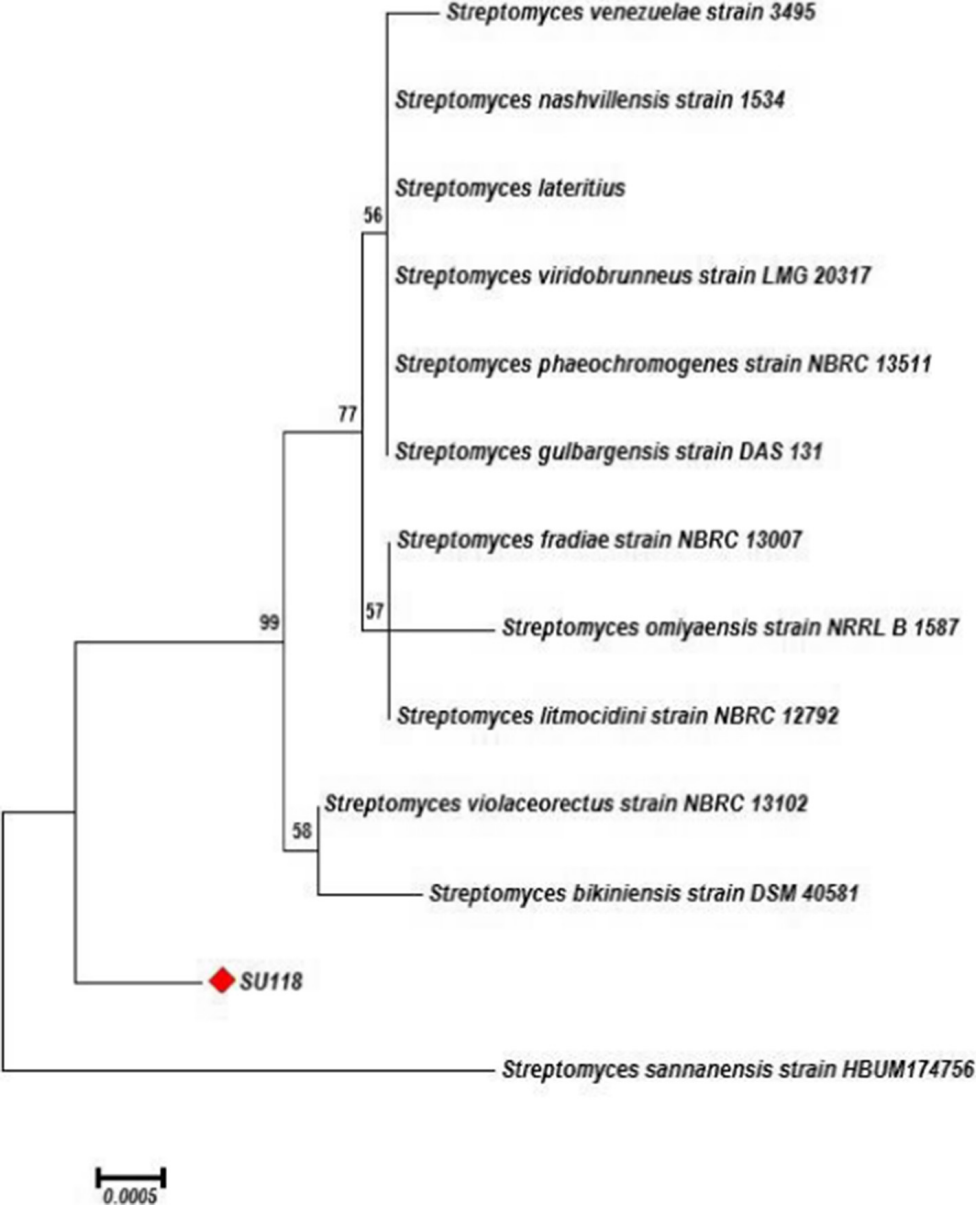
**Phylogenetic tree based on 16S rRNA gene sequences from the strain SU118 and other** ***Streptomyces*** **species.**

The actinomycetes strain SU118 isolated from soil samples of *phoomdi* in Loktak Lake of Manipur, India is a Gram-positive filamentous bacterium. The vegetative mycelium showed cream-light brown colour while the aerial mycelium showed light gray colour. The culture, when examined by light microscope (100 ×) have straight to flexuous sporophores arising from the aerial mycelium and may be placed in the Rectus-Flexibilis (RF) group of *Streptomyces* species [35]. The strain SU118 could grow up to 40°C and pH 9 with 3% NaCl concentration. LL-diaminopimelic acid (L-DAP) was present in the cell wall but no characteristic sugar. The strain SU118 could utilize glucose, arabinose, mannitol, xylose, meso-inositol, raffinose, rhamnose, salicin, sucrose, galactose and fructose as the carbon source without the production of acid. The morphological, physiological and biochemical characteristics of the strain SU118 is shown in Table 1. The 16S rRNA gene sequence of the strain SU118 was compared with the nucleotide sequences of other *Streptomyces* strains from the NCBI GenBank database. The phylogenetic tree generated on the basis of 16S rRNA gene sequence of the strain SU118 and the nucleotide sequences from closely related *Streptomyces* strains using neighbor-joining method is presented in Figure 1. The strain has got maximum 16S rRNA gene sequence homology (99%) with *S. sannanensis* strain HBUM174756. Therefore, on the basis of morphological, physiological, biochemical and analysis of the 16S rRNA gene sequence, the new isolate SU118 was designated as *S. sannanensis* strain SU118. The 16S rRNA gene partial sequence of the isolate SU118 has been deposited in the GenBank database under the accession number EU278596. The phylogenetic tree of the strain SU118 has been deposited in TreeBASE repository with the submission ID 16506. Further the information associated with the phylogenetic placement of the strain SU118 is available in http://treebase.org/treebase-web/search/study/summary.html?id=16506. The identity of the isolate was further confirmed by the Microbial Type Culture Collection and Gene Bank (MTCC) of the Institute of Microbial Technology, Chandigarh, India. The culture has been deposited in MTCC, an International Depository Authority and the accorded accession number is MTCC-7041.

**Table 1:** **Morphological, physiological and biochemical characteristics of** ***Streptomyces sannanensis*** **SU118**

**Properties**	**Result**
**Morphological characteristics**	
Cell shape	Mycelial
Sporophore morphology	Rectus- Flexibilis (RF)
Aerial mycelium colour	Light gray
Substrate mycelium colour	Cream-light brown
**Physiological characteristics**	
Growth under anaerobic condition	- ve
Acid-fast reaction	- ve
Production of melanoid pigment	+ ve
Temperature range for growth	25 – 40°C
Optimum temperature for growth	28°C
pH range for growth	5 - 9
Optimum pH for growth	7
Growth on Mc Conkey agar	- ve
NaCl tolerance	3%
**Biochemical characteristics**	
Gram reaction	+ ve
Catalase production	- ve
Oxidase production	- ve
Urease production	- ve
Hydrogen sulfide production	- ve
Nitrate reduction	- ve
Gelatin liquefaction	- ve
Methyl red test	- ve
Vogues proskauer test	- ve
Indole production test	- ve
Citrate utilization test	- ve
Starch hydrolysis	+ ve
Casein hydrolysis	- ve
**Acid production from**	
Glucose	- ve
Arabinose	- ve
Mannitol	- ve
Xylose	- ve
Meso-inositol	- ve
Raffinose	- ve
Rhamnose	- ve
Salicin	- ve
Sucrose	- ve
Galactose	- ve
Fructose	- ve
Cell-wall amino acids	LL-Diaminopimelic aid
Cell-wall sugars	No diagnostic

Note: + ve: Positive for the test; − ve: Negative for the test.

### Antimicrobial potential of the strain SU118

**Figure 2:**
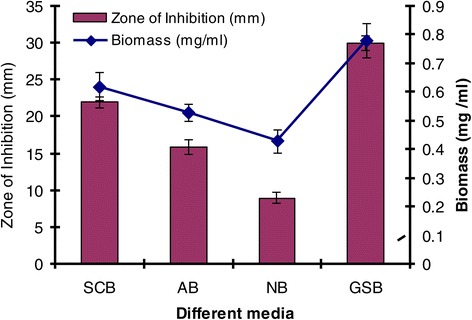
**Effect of various culture media on growth and antimicrobial agent production by** ***Streptomyces sannanensis*** **SU118 (SCB- Starch casein broth; AB- Actinomycete broth; NB- Nutrient broth; GSB- Glucose soyabean meal broth).**

During the course of screening of actinomycetes possessing antimicrobial activity, it was observed that *S. sannanensis* SU118 was highly antagonist to Gram-positive bacteria including one clinical isolate of *S. aureus* while it does not have any inhibitory effect against any of the tested Gram-negative bacteria (Table 2). *S. aureus* MTCC 96 was the most sensitive to the antimicrobial agent; hence it was taken as test organism for further studies. Among the different liquid media studied for antimicrobial agent production in shake flask condition, GSB was found to be most suitable for growth as well as synthesis of antimicrobial agent by *S. sannanensis* SU118 (Figure 2). It was observed that the culture filtrate of *S. sannanensis* SU118 grown on GSB exhibited maximum inhibition zone diameter against *S. aureus* MTCC 96 followed by the culture filtrate grown on SC broth, while lowest activity was seen with the one grown on NB.

**Table 2:** **Inhibition zone diameter and minimum inhibitory concentration of the active compound (R**_**f**_ **value 0.56) produced by** ***Streptomyces sannanensis*** **SU118**

**Test microorganisms**	**Inhibition zone diameter (mm) ¥**	**MIC (μg/ml)**	**MIC of penicillin (μg/ml)**
*Bacillus subtilis* MTCC 736	25 ± 1.0	2.0	0.5
*Bacillus circulans* MTCC 8074	23 ± 1.2	3.0	0.5
*Bacillus megaterium* MTCC 8075	26 ± 1.5	2.0	0.5
*Staphylococcus aureus* MTCC 96	31 ± 0.9	0.5	0.25
*Staphylococcus aureus* (Clinical isolate)	30 ± 0.9	0.5	0.25
*Micrococcus luteus* MTCC 2987	25 ± 1.3	2.0	0.5
*Mycobacterium phlei* MTCC 1724	25 ± 1.0	2.0	0.5
*Mycobacterium smegmatis* MTCC 6	21 ± 0.5	3.0	1.0
*Escherichia coli* MTCC 739		ND	ND
*Klebsiela pneumoniae* MTCC 3040		ND	ND
*Pseudomonas aeruginosa* MTCC 2453		ND	ND

Note: Data express as mean ± SD (n =3) “ – ” : No Inhibition Zone; ¥ Concentration of the active compound: 1 mg/ml; ND – Not determined.

### Effect of cultural parameters on growth and production of antimicrobial agent

**Figure 3:**
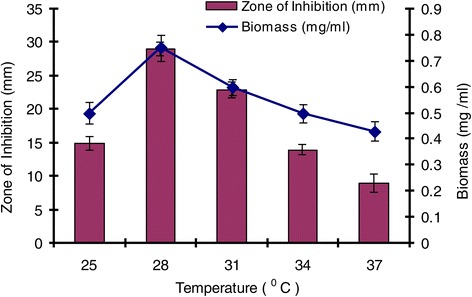
**Effect of incubation temperature on growth and antimicrobial agent production by** ***Streptomyces sannanensis*** **SU118.**

**Figure 4:**
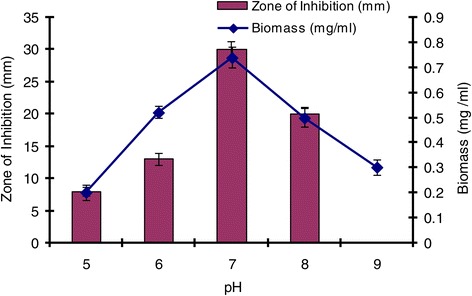
**Effect of pH on growth and antimicrobial agent production by** ***Streptomyces sannanensis*** **SU118.**

**Figure 5:**
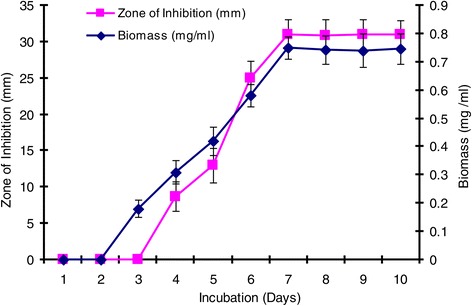
**Effect of incubation period on growth and antimicrobial agent production by** ***Streptomyces sannanensis*** **SU118.**

**Figure 6:**
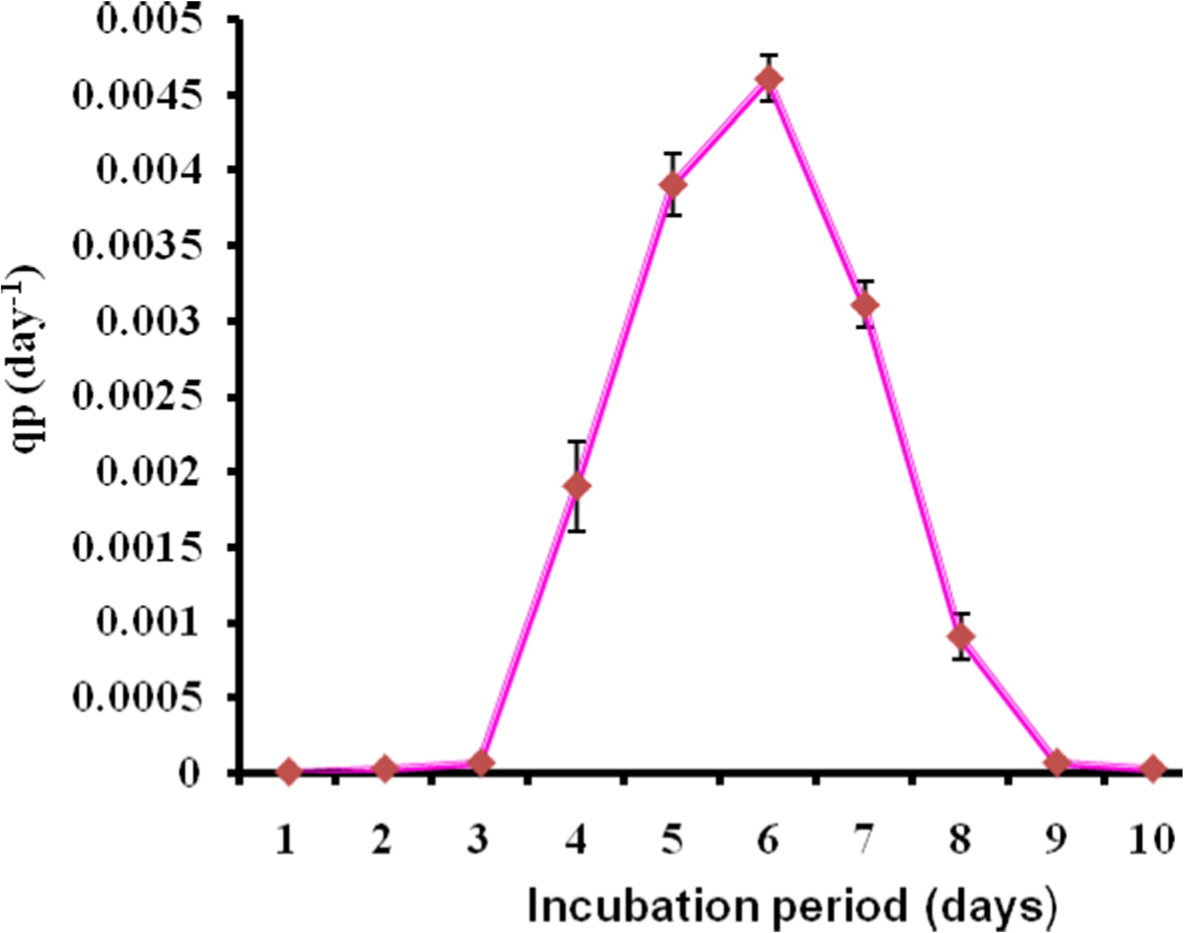
**Specific rate of antimicrobial agent production (qp) by** ***Streptomyces sannanensis*** **SU118** ***.***

The environmental requirements and cultural conditions for growth and production of antimicrobial agent by *S. sannanensis* SU118 has been studied on GSB in shake flask condition. The strain *S. sannanensis* SU118 showed a narrow range of incubation temperature for relatively good growth and antibiotic production. 28°C was found to be optimum for highest growth as well as maximum antimicrobial agent production by the strain (Figure 3). In terms of its optimum temperature for growth, the organism appeared to be mesophilic in nature. The highest growth as well as maximum antimicrobial agent production was obtained at pH 7. Poor growth was evident at pH values below and above neutral (Figure 4). Incubation period up to seven days was found to be optimum for maximum growth as well as antimicrobial agent production by *S. sannanensis* SU118 (Figure 5). The highest value of specific rate of production of the antimicrobial agent (0.0046 day ^−^1) was observed on 6^th^ day (Figure 6).

### Purification and bioactivity of the metabolite

**Figure 7:**
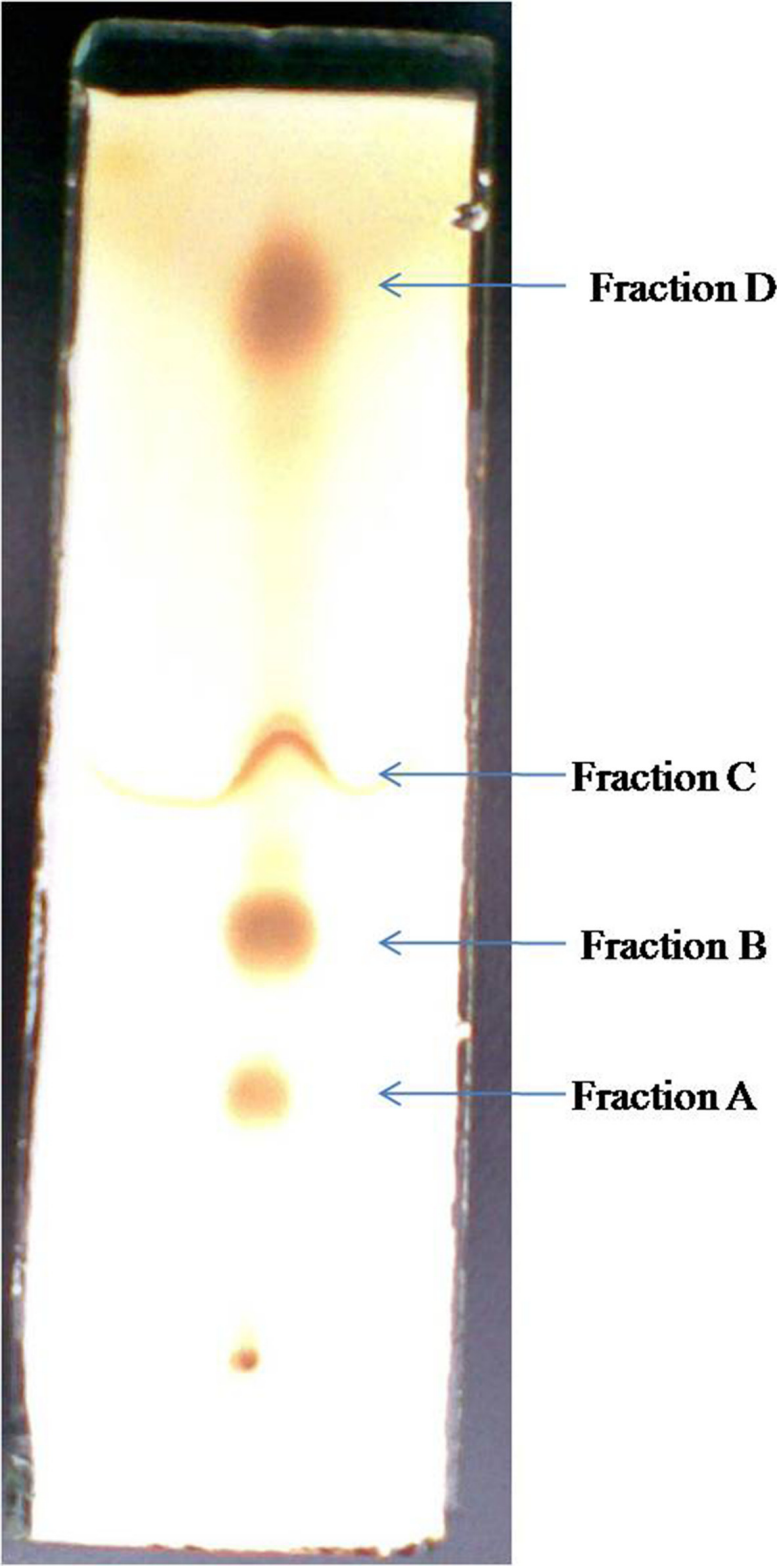
**Thin layer chromatography plate showing the separation of bioactive compound (fraction B) obtained from** ***Streptomyces sannanensis*** **SU118.**

The dark brown colored crude metabolite recovered by solvent extraction method using ethyl acetate was purified by TLC in a running solvent system of hexane and ethyl acetate in 1:3 ratio. Four fractions designated as A, B, C and D (Figure 7) with different R_f_ values recovered from TLC plates were dissolved in 10% DMSO and bioassayed against the test microorganisms. The fraction B with R_f_ value 0.56 and UV λ_max_ 275.0 nm in ethyl acetate exhibits antimicrobial activity against all the Gram-positive bacteria tested while it does not affect Gram-negative bacteria (Table 2). Other fractions (A, C and D) did not show any activity against the test organisms. Hence the fraction B is considered as the active metabolite. The results indicated that *S. aureus* MTCC 96 was the most sensitive while *M. smegmatis* MTCC 6 was found to be least sensitive to the active metabolite obtained from *S. sannanensis* strain SU118. The quantitative efficacy of the active metabolite was estimated as MIC and it has got lowest MIC (0.5 μg/ml) against *S. aureus* MTCC 96 and *S. aureus* (Clinical isolate), whereas highest (3.0 μg/ml) was recorded against *M. smegmatis* MTCC 6 and *B. circulans* MTCC 8074 (Table 2). The active metabolite was soluble in ethyl acetate, hexane, chloroform and DMSO whereas sparingly soluble in water.

### Determination of rate of kill

The results of time-kill assay are presented in Table 3. Data are presented in terms of the log_10_ cfu/ml change and are based on the conventional bactericidal activity standard, that is, a 3 log_10_ cfu/ml or greater reduction in the viable colony count relative to the initial inoculums according to Pankey and Sabath [36]. After 4 h of incubating the bacteria with the 1 × MICs and 2 × MICs, the average log reduction in the viable cell count ranged between 0.198 log_10_ and 1.218 log_10_ cfu/ml. After 8 h of incubation with these different concentrations, the average log reduction in the viable cell count ranged between −2.075 log_10_ and 0.891 log_10_ cfu/ml. The incubation of the bacteria for 4 h at 1 × MICs resulted in the average log reduction of the viable cell count ranging between 0.959 log_10_ and 1.218 log_10_ cfu/ml, while after 8 h of incubation, the average log reduction ranged between 0.367 log_10_ and 0.891 log_10_ cfu/ml. The incubation of the bacteria for 4 h at 2 × MICs resulted in the average log reduction of the viable cell count ranging between 0.198 log_10_ and 0.487 log_10_ cfu/ml, while after 8 h of incubation, the average log reduction in the viable cell count ranged between −2.075 log_10_ and −1.484 log_10_ cfu/ml. The greatest log reduction in cell density with the antimicrobial agent was observed in *S. aureus* MTCC 96 (−2.075 log_10_ cfu/ml) followed by *S. aureus* (Clinical isolate) (−1.953 log_10_ cfu/ml), while least log reduction in cell density was observed in *M. smegmatis* MTCC 6 (−1.484 log_10_ cfu/ml).

**Table 3:** ***In vitro*** **time-kill assessment of the antimicrobial agent against the test microorganisms**

**Test bacterial strains**	**Log**_**10**_ **Kill**			**Log**_**10**_ **Kill**			**Log**_**10**_ **Kill**		
	**(½) X MIC**			**MIC**			**2 X MIC**		
	**0 h**	**4 h**	**8 h**	**0 h**	**4 h**	**8 h**	**0 h**	**4 h**	**8 h**
*Bacillus subtilis* MTCC 736	2.381	3.131	4.213	2.364	1.165	0.507	2.286	0.365	−1.776
*Bacillus circulans* MTCC 8074	2.456	3.233	4.336	2.478	1.213	0.578	2.346	0.389	−1.501
*Bacillus megaterium* MTCC 8075	2.234	3.691	4.821	2.227	1.186	0.515	2.242	0.296	−1.875
*Staphylococcus aureus* MTCC 96	2.487	2.756	3.251	2.491	0.959	0.367	2.475	0.198	−2.075
*Staphylococcus aureus* (Clinical isolate)	2.983	3.423	4.206	2.907	0.979	0.406	2.915	0.203	−1.953
*Micrococcus luteus* MTCC 2987	2.169	2.792	3.042	2.127	1.146	0.723	2.217	0.401	−1.637
*Mycobacterium phlei* MTCC 1724	2.291	2.813	3.112	2.251	1.187	0.811	2.252	0.431	−1.576
*Mycobacterium smegmatis* MTCC 6	2.386	2.912	3.102	2.298	1.218	0.891	2.379	0.487	−1.484

## Discussion

Actinomycetes have been recognized as the potential producers of metabolites such as antibiotics, growth promoting substances for plants and animals, immunomodifiers, enzyme inhibitors and many other compounds of use to man. Isolation of new microbial species from hitherto unexplored areas and/or from extreme environments is one of the more efficient approaches for the development of potential novel bioactive metabolites [37,38]. The high proportion of strains producing antimicrobials from such environments may be associated with defensive or aggressive roles of the organisms for maintaining their ecological niche in such environments. Further, the thriving of microorganisms in such competitive environments is assumed that their metabolic compatibility is strongly influenced by natural selection [39]. With this perspective, an antimicrobial agent producing actinomycete strain SU118 was isolated from the *phoomdi* soil of Loktak lake in Manipur, India. The isolate has been characterized and identified as *S. sannanensis* strain SU118.

The antimicrobial activity of *S. sannanensis* SU118 was screened against various Gram-positive and Gram-negative organisms. The results showed that *S. sannanensis* SU118 secretes a narrow spectrum antimicrobial agent which could inhibit the growth of only Gram-positive bacteria. However, it did not affect the growth of Gram-negative organisms. Similar antimicrobial activity specific to Gram-positive bacteria has also been reported in *S. aburaviensis* strain Kut-8 [40]. The reason for differential sensitivity between Gram-positive and Gram-negative bacteria could be ascribed to the morphological differences between these microorganisms; Gram-negative bacteria have an outer polysaccharide membrane carrying the structural lipopolysaccharide components. This makes the cell wall impermeable to lipophilic compounds; the Gram-positive bacteria, on the other hand, will be more susceptible as they have only an outer peptidoglycan layer which is not an effective permeability barrier [41].

The study on the production of antimicrobial agent usually involves a search of a suitable culture medium. Among the four media tried, GSB was found to be the optimum medium for antimicrobial agent production by *S. sannanensis* SU118. It was evident from the finding that the antimicrobial agent production by *S. sannanensis* SU118 was positively affected by the nature and type of carbon and nitrogen sources in the medium. The results showed that antimicrobial agent production was higher in medium having glucose and soyabean meal as carbon and nitrogen source respectively. This result is quite comparable with *S. griseocarneus*, for which glucose was found to be suitable carbon source for the antibiotic production [42]. Considering the carbon source, simple sugar such as glucose, fructose, sucrose as sole carbon source enhanced growth as well as bioactive metabolite production rather than more complex carbons [43]. Our result is also in conformity with the findings of Singh *et al*. [7] for which soyabean meal proved to be suitable carbon source for antibiotic production by *S. tanashiensis* A2D. The results indicated the dependence of the antimicrobial agent synthesis on the medium constituents. In fact, it has been shown that the nature of carbon and nitrogen sources strongly affect antibiotic production in different organisms [44]. The strain *S. sannanensis* SU118 showed a narrow range of incubation temperature for relatively good growth and antimicrobial agent production. Highest growth and antimicrobial agent production was obtained at 28°C. The temperature range adequate for good production of secondary metabolites is narrow, for example, 5 ~ 10 degrees [45]. The maximum growth as well as highest antimicrobial activity by *S. sannanensis* SU118 was achieved at pH 7 while it does not exhibit any activity at pH 9. There are reports regarding the role of pH on the production of bioactive metabolite by microorganisms [46]. Incubation period upto seven days was found to be optimum for highest antimicrobial agent production by the strain SU118. Griffiths and Saker [47] reported that maximum secretion of bioactive metabolites by *Cylindrospermopsis raciboskii* was found to occur as cultures moved into the post-exponential phase of growth, while Egorov [48] postulated that maximum antimicrobial activity was attained after reaching the maximum value of the biomass. The results indicated that environmental factors and cultural conditions like incubation temperature, pH and incubation period were found to have profound influence on antimicrobial agent production by *S. sannanensis* SU118 as surveyed in streptomycetes by other investigator [49]. The result for the determination of specific rate of production of antimicrobial agent by the strain SU118 showed to be maximum on the sixth day (0.0046 day ^−^1). The strain SU118 showed specific potential in antimicrobial agent production. Other observations on the specific rate of production of bioactive metabolite by different microorganism have also been reported [50,51].

The solvent extraction of the culture broth of *S. sannanensis* SU118 using ethyl acetate and subsequent purification led to the recovery of a potent antibacterial agent active against Gram-positive bacteria. Remya and Vijaykumar [52] have reported the antimicrobial potential of compound obtained from ethyl acetate extract of *Streptomyces* strain RM42. Similarly, the extraction of antibiotics has been carried out from streptomycetes by using various solvents including ethyl acetate and methanol [53,54].

The resultant effect of incubating the bacteria at 2 × MICs was a significantly rapid reduction in the average log of the viable cells counts. This reduction is greater than the rate of kill observed in the test bacterial strains treated with the 1 *×* MICs. The significant reduction in the cell counts between 4 and 8 h of incubation period acknowledged the fact that the antimicrobial agent was highly bactericidal seeing that the bacterial colonies were almost totally wiped out after incubating for 8 h. On the contrary, there was a net growth of all the test bacterial strains treated with the 1/2 × MICs of the antimicrobial agent. The growth inhibition and efficacy of the antimicrobial agent were observed to be concentration and time dependent producing distinct time-kill profiles for the tested bacterial strains.

Detection and identification of members of the genus *Streptomyces* are of great value because they provide a rich source of antibiotics. The emergence and dissemination of antibacterial resistance is well documented as a serious problem worldwide [55]. Smith *et al*. express that “The emergence of bacterial resistance threatens to return us to the era before the development of antibiotics” [56]. The perspective of rapid emergence of drug resistance among bacterial pathogens shows that the potencies of prevalent antibiotics are decreasing steadily, leading to reduced useful-period of drugs. This situation compounds the need for the investigation of new, safe and effective antimicrobials for replacement with invalidated antimicrobials or use in antibiotic rotation programs [57].

## Conclusions

The strain SU118 showed a narrow spectrum of activity inhibiting only Gram-positive bacteria, when tested with crude culture filtrate and also with active metabolite (R_f_ value = 0.56). The MICs of the antimicrobial agent against the test pathogens indicated the potent activity which highlights its prospective and could be a candidate in the generation of new antimicrobial agents and also throws a light towards the fight against drug resistant pathogens especially of the methicillin resistant *Staphylococcus aureus* (MRSA), since this particular isolate SU118 inhibits only the Gram positive bacteria. The findings of the present study showed that naturally occurring actinomycetes have a great potential to produce metabolite active against bacteria enabling discovery of new antibiotics and hence merit future studies. Further ongoing detailed characterization and structure elucidation of the bioactive metabolite may be a new entity reported from this unique untapped ecological niche.
